# Expert guidance on prophylaxis and treatment of dermatologic adverse events with Tumor Treating Fields (TTFields) therapy in the thoracic region

**DOI:** 10.3389/fonc.2022.975473

**Published:** 2023-01-04

**Authors:** Milan J. Anadkat, Mario Lacouture, Adam Friedman, Zachary D. Horne, Jae Jung, Benjamin Kaffenberger, Sujith Kalmadi, Liza Ovington, Rupesh Kotecha, Huda Ismail Abdullah, Federica Grosso

**Affiliations:** ^1^ Division of Dermatology, Department of Medicine, Washington University, St. Louis, MO, United States; ^2^ Dermatology Service, Department of Medicine, Memorial Sloan Kettering Cancer Center, New York, NY, United States; ^3^ Division of Dermatology, Department of Medicine, George Washington University School of Medicine and Health Sciences, Washington, DC, United States; ^4^ Department of Radiation Oncology, Allegheny Health Network Cancer Institute, Pittsburgh, PA, United States; ^5^ Department of Dermatology, Norton Healthcare, Louisville, KY, United States; ^6^ Wexner Medical Center, Ohio State University, Columbus, OH, United States; ^7^ Oncology and Haematology Department, Ironwood Cancer & Research Center, Chandler, AZ, United States; ^8^ Ovington & Associates, Walnutport, PA, United States; ^9^ Miami Cancer Institute, Baptist Health South Florida, Miami, FL, United States; ^10^ Novocure Inc., New York, NY, United States; ^11^ Mesothelioma Unit, SS Antonio e Biagio General Hospital, Alessandria, Italy

**Keywords:** Tumor Treating Fields, TTFields, skin management, thoracic oncology, skin adverse events

## Abstract

Tumor Treating Fields (TTFields) are electric fields, delivered via wearable arrays placed on or near the tumor site, that exert physical forces to disrupt cellular processes critical for cancer cell viability and tumor progression. As a first-in-class treatment, TTFields therapy is approved for use in newly diagnosed glioblastoma, recurrent glioblastoma, and pleural mesothelioma. Additionally, TTFields therapy is being investigated in non-small cell lung cancer (NSCLC), brain metastases from NSCLC, pancreatic cancer, ovarian cancer, hepatocellular carcinoma, and gastric adenocarcinoma. Because TTFields therapy is well tolerated and delivery is locoregional, there is low risk of additive systemic adverse events (AEs) when used with other cancer treatment modalities. The most common AE associated with TTFields therapy is mild-to-moderate skin events, which can be treated with topical agents and may be managed without significant treatment interruptions. Currently, there are no guidelines for oncologists regarding the management of TTFields therapy-related skin AEs in the thoracic region, applicable for patients with pleural mesothelioma or NSCLC. This publication aims to provide guidance on preventing, minimizing, and managing dermatologic AEs in the thoracic region to help improve patient quality of life and reduce treatment interruptions that may impact outcomes with TTFields therapy.

## Introduction

### Introduction to Tumor Treating Fields therapy

**Figure 1:**
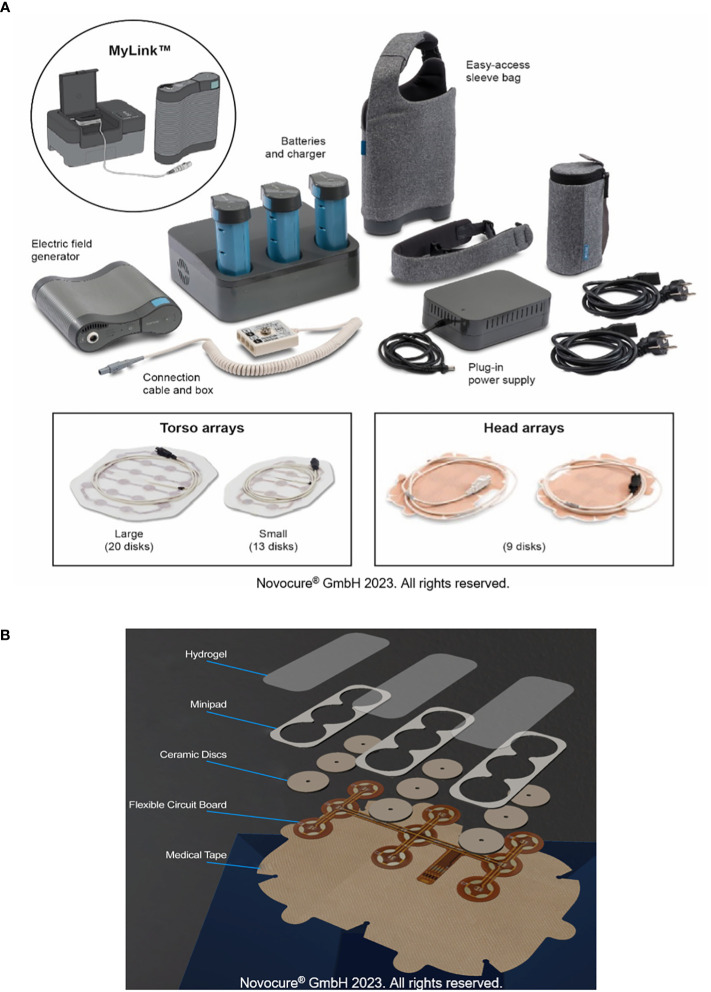
TTFields therapy device. All components of the portable medical device that generates TTFields **(A)**. Components of the arrays used to deliver TTFields therapy **(B)**. *TTFields, Tumor Treating Fields*.

Tumor Treating Fields (TTFields) are electric fields that exert physical forces to disrupt cellular processes critical for cancer cell viability and tumor progression (1). As a locoregional treatment, TTFields therapy is noninvasive as the electric fields are continuously generated by a portable medical device, and delivered via arrays placed directly at the surface of the skin at the site of the tumor (**Figure 1**) (2). Currently, TTFields therapy is approved for the treatment of adult patients with unresectable pleural mesothelioma (150 kHz), and newly-diagnosed or recurrent glioblastoma (200 kHz) (3–5) based on safety data from the STELLAR study, and safety and efficacy data from the EF-11 and EF-14 studies (6–10). Median overall survival (OS) was 18.2 months in patients with pleural mesothelioma receiving TTFields therapy with pemetrexed plus platinum therapy (6, 7). Furthermore, in a phase I/II study of TTFields therapy with pemetrexed in patients with recurrent stage IIIB and stage IV non-small cell lung cancer (NSCLC), median OS was 13.8 months and the 1-year survival rate was 57% (11). Use of TTFields therapy with immune checkpoint inibihitors or docetaxel in patients with advanced NSCLC following platinum failure is also being studied in the ongoing phase III LUNAR study (NCT02973789). The safety and efficacy of TTFields therapy in other solid tumors, including pancreatic cancer (12), hepatocellular carcinoma (13), ovarian cancer (14), gastric adenocarcinoma (15), and brain metastases from NSCLC (16) is also being investigated in ongoing clinical studies.

The most common adverse events (AEs) associated with TTFields therapy are dermatologic AEs, as reported in both clinical studies and recent real-world data (7, 9–12, 14, 17). Advice on the prevention and management of dermatologic scalp AEs associated with TTFields therapy are available; however, no such guidance exists for the thoracic region, which is especially needed as skin AEs in the thoracic region are often exacerbated from the motion of respiration (18). The objective of this publication is to provide practicing oncologists with robust and clear practical symptom-based guidance to minimize, prevent, and manage localized TTFields therapy-related AEs in the thoracic region. The guidance presented here is based on the authors’ overall clinical experience and consensus regarding skin wellness strategies, and is not supported by clinical study data from a controlled setting.

### Burden of skin AEs

Skin AEs occur as a side effect of many systemic and localized treatments commonly used for cancers including NSCLC and pleural mesothelioma. While these are well-recognized by healthcare professionals, the economic and personal burden of skin conditions and AEs can be high, therefore affecting mental, physical, and emotional well-being, and potentially having a significantly detrimental impact on quality of life.

Certain therapies increase the risk of developing skin AEs more than others. For example, radiation therapy induces dermatitis, which can be erosive or ulcerative, and chemotherapies can damage hair, skin, and nails, due to their cytotoxic nature (19, 20). Furthermore, immunotherapies can instigate immune-related AEs, including psoriasiform, lichenoid, and blistering dermatitis (20, 21), or other skin AEs, including rash, skin thickening, and itching (22–24).

Importantly, the occurrence of skin AEs can disrupt treatment regimens as patients may need to interrupt or potentially discontinue treatment until the AE is completely resolved. Skin AEs may also impact on the practical aspects of treatment administration (e.g., adhesion of the arrays to the skin). Taken together, the impact on quality of life, treatment, and associated economic burden supports the need for practical prevention and management strategies for dermatologic AEs. Therefore, the management of skin AEs resulting from cancer therapies is of paramount importance and represents a challenge for oncologists and dermatologists.

### TTFields therapy-associated skin AEs

**Figure 2:**
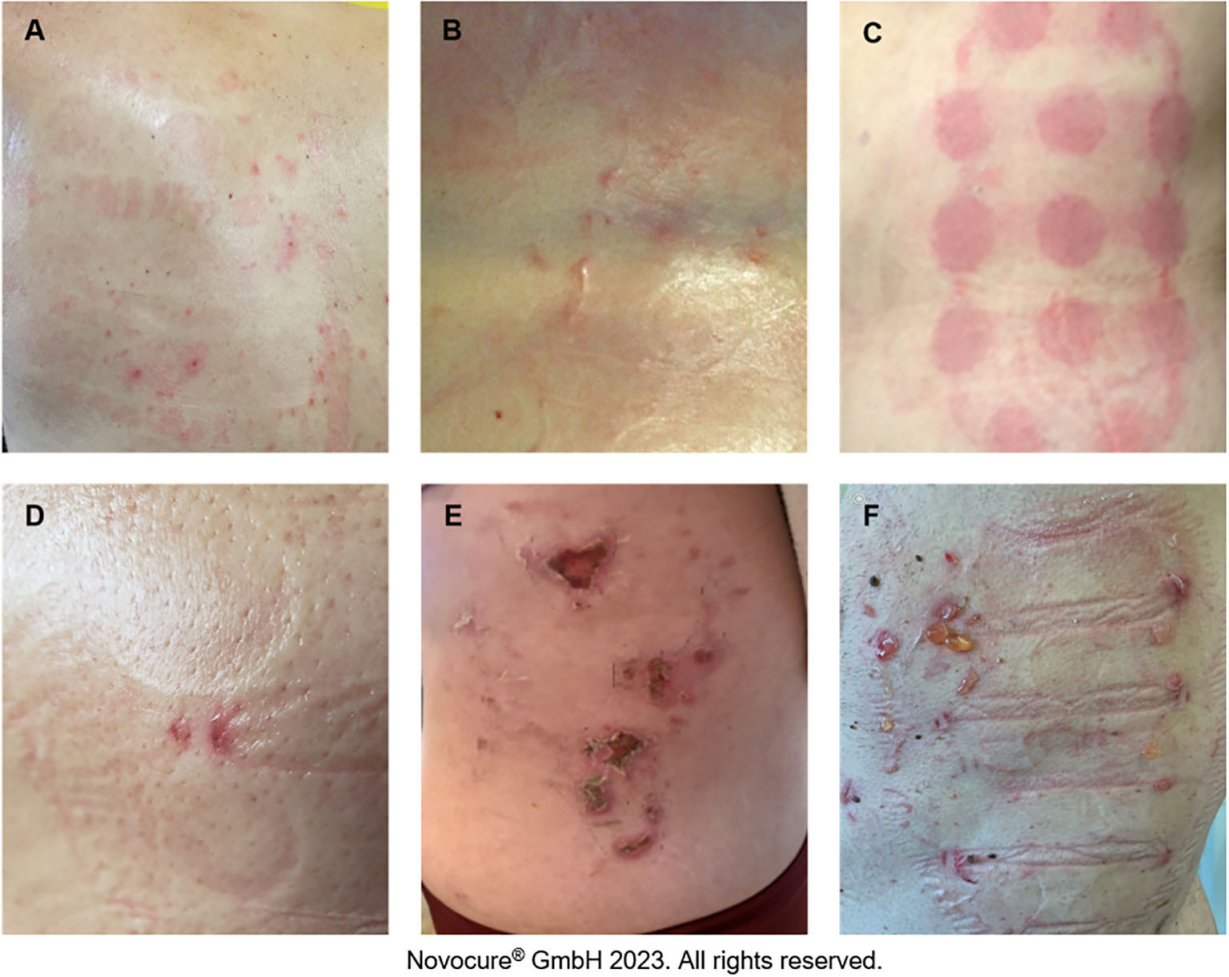
Potential TTFields therapy-associated skin AEs. Pruritus **(A)**, hyperhidrosis **(B)**, contact dermatitis **(C)**, skin erosion **(D)**, pressure necrosis **(E)**, contact dermatitis and infection **(F)**.

AEs associated with TTFields therapy are primarily dermatologic and are typically observed where the skin is in direct contact with the adhesive or hydrogel from the array (25). The most common types are contact dermatitis, pruritus, hyperhidrosis, pressure necrosis, and skin erosions (**Figure 2**; **Supplementary Figure S1**). Findings from clinical studies have demonstrated that most skin AEs are mild and manageable without the need to significantly disrupt treatment (3, 4, 10), which is supported by real-world data (17, 26). Although analysis of skin AEs from pilot studies has not identified any correlation between select risk factors and the occurrence of TTFields therapy-related skin AEs (**Table 1**), several factors may increase the risk and severity of skin damage. Such risk factors could be innate or environmental and can be loosely grouped into three categories: product-related, medical-related, and patient-related.

**Table 1:** Overview of skin AEs from pivotal TTFields therapy clinical studies.

Study	Indication	Patients, n	Patients with any gradeskin AEs, n (%)	Patients with grade 3^a^ skin AEs, n (%)
EF-11 (NCT00379470) (10)	rGBM	116	19 (16)	0
EF-14 (NCT00916409) (9)	ndGBM	456	237 (52)	9 (2)
EF-15 (NCT00749346) (11)	NSCLC	42	18 (43)	1 (2)
STELLAR (NCT02397928) (7)	Pleural mesothelioma	80	57 (71)	4 (5)
PANOVA (NCT01971281) (12)	Pancreatic cancer	40	21 (53)	7 (18)
INNOVATE (NCT02244502) (14)	Recurrent ovarian carcinoma	31	28 (90)	2 (6)
HEPANOVA (NCT03606590) (13)	Hepatocellular carcinoma	27	19 (70)	1 (4)

AEs, adverse events; GBM, glioblastoma; nd, newly diagnosed; NSCLC, non-small cell lung cancer; r, recurrent; TTFields, Tumor Treating Fields.

a Grade 3 events are severe but not immediately life-threatening, as per CTCAE v5.0 (27).

Copyright Kutuk T et al. Cancers 2022. Adapted and reproduced under the terms of the Creative Commons Attribution License (CC BY).

Given that TTFields are delivered using skin-adhered arrays, there is an inherent risk of skin irritation/damage. Ingredients in the hydrogel used on the arrays can cause contact dermatitis and pruritus; frictional contact dermatitis and pressure necrosis can occur either due to shear forces from routine array replacement and/or rubbing between the thorax and the device itself (particularly the flexible circuit board, **Figure 1**), as a result of normal movement of the body, or mechanical pressure from the device hardware (**Supplementary Figure S1**). Improper management of early injury can evolve into areas of skin erosions and pressure necrosis as secondary events, caused by ineffective management of less severe AEs (**Supplementary Figure S1**). These cutaneous AEs display an anatomic predilection based on the degree of movement of the skin and the size of the array used. It should be noted that whilst skin burns can occur as a result of using wearable medical devices, such as transcranial direct current stimulation (28), there are no such reports in patients receiving TTFields therapy.

Medical-related risk factors include prior treatment or concomitant therapies such as radiotherapy and chemotherapy, which can damage or weaken the skin. Radiotherapy delivery technique and target location may lead to a higher risk of dermatologic AEs when applied concurrently with TTFields therapy. Preliminary data extrapolated from glioblastoma experience suggests that the two treatments can be utilized with no moderate-severe skin toxicities reported (29, 30). In the Phase I pilot study examining scalp-sparing radiation with concurrent temozolomide and TTFields therapy in patients with newly diagnosed glioblastoma, there were no Grade ≥3 AEs (per Common Terminology Criteria for Adverse Events [CTCAE] version 5.0) or discontinuations due to dermatologic AEs (27, 30). When treating patients with pleural mesothelioma, radiation field sizes tend to be larger with targets closer to the skin, and therefore skin doses may be higher, leading to a higher incidence of radiation dermatitis. Chemotherapy delivered concomitantly with TTFields therapy and radiation may further degrade the integrity of the skin and lead to higher rates of dermatologic AEs.

Possible innate risk factors for developing skin AEs can include older age, genetics, sex, allergies, history of skin disorders, skin health, extent of scar tissue, and existing photodamage (31–33). Scar tissue is weaker and less flexible than healthy skin, which may increase the risk of developing AEs. Furthermore, baseline moisturization level of the skin is another factor that can influence risk of skin irritation, since dehydrated skin is thinner, less elastic, and more susceptible to irritation (physical and molecular), whereas hydrated skin is more resilient and heals more readily (34).

Given that patients receiving TTFields therapy may experience dermatologic AEs, and more severe AEs are preceded by low-grade AEs (35), and since no specific prognostic factors for development of TTFields therapy-related skin AEs have been identified, a pragmatic approach should be adopted for all patients considering TTFields therapy.

### Overview of skin structure

Skin characteristics (such as thickness, follicle density, and adipocyte density) naturally vary from person-to-person and by body location (**Table 2**). As such, it is important to understand how skin structure and characteristics affect the inherent strength, resilience, and healing abilities of the skin, so that the risk to a given patient of developing treatment-associated AEs can be assessed and planned for accordingly.

**Table 2:** Skin characteristics by body area.

Characteristic	Scalp	Abdomen	Back	Thorax^a^
**Mean skin thickness, μm**
Epidermis Dermis Total	111 2238 2351	127 4550 4680	140 3265 3575	77–164 4717–5888 4794–6052
Hair follicle density	++	±		Variable
Radiotherapy effects	+	++		+
Elasticity	–	++		++
Eccrine sweat	++	+		+
Sebaceous glands	++	+		+
Covering/clothing	–	+++		+++

a There are differences in the thoracic region between sexes.

–, absent/minimal; +, present; ++, abundant; +++, very abundant.

Data are based on authors’ experience and also from Oltulu P et al. Turk J Plastic Surg 2018 (40).

Areas of skin with high follicle density, such as the scalp, typically heal faster than those with lower densities (33, 36, 37). The same is true for regions with a high concentration of adipocytes and sebocytes, such as the thighs or buttocks where there is a high amount of subcutaneous adipose tissue underlying the dermis (33, 37–39). Although the dermis and epidermis in the thoracic region are considerably thicker compared with the scalp (40), safety outcomes generally demonstrate a higher incidence of TTFields therapy-related skin AEs with the arrays placed on the thoracic region versus the scalp region (**Table 1**).

## Guidance to prevent and minimize skin AEs associated with TTFields therapy

### Practical guidance for preventing and minimizing dermatologic AEs

#### Maintaining optimal skin health

**Figure 3:**
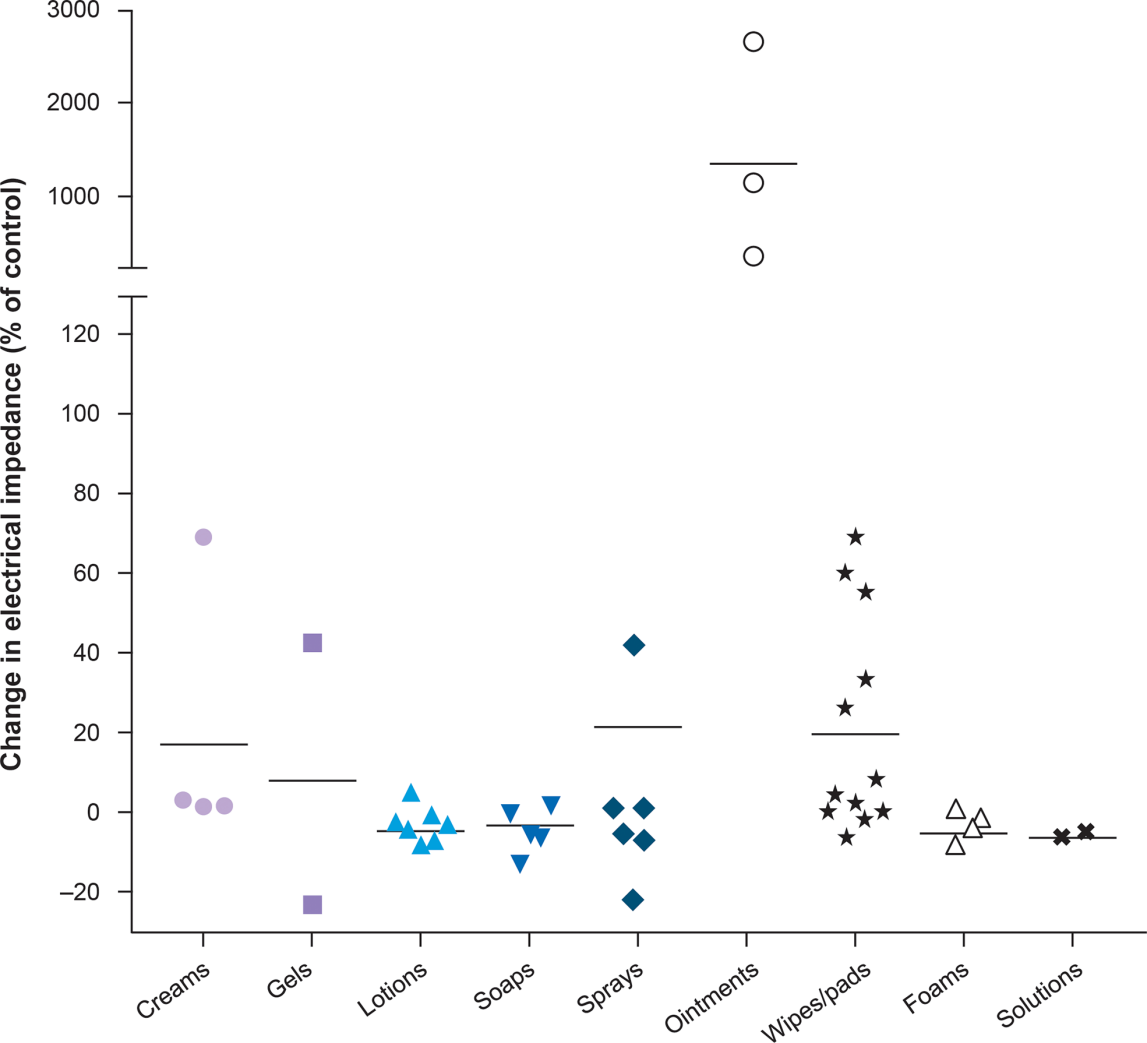
Topical agents tested with TTFields therapy on the scalp (25). Horizontal bars represent the mean for each category. Copyright Lacouture ME et al. *Front Oncol* 2020. Adapted and reproduced under the terms of the Creative Commons Attribution License (CC BY).

Maintaining optimal skin health is fundamental to prevent skin AEs. The area of the skin on which arrays will be placed should be kept clean and dry, and routine cleaning of the skin should be performed using water. The skin should be moisturized regularly with mild products that are compatible with TTFields therapy, meaning they will not impact electrical impedance, to ensure hydration, strengthen the skin barrier, and promote healing in the event of damage. The moisturizer Topic Medic Body Lotion has been tested and shows minimal impact on electrical impedance (25). Various skin care formulations have shown differing effects on electrical impedance (**Figure 3**).

#### Optimizing skin preparation

##### Hair removal

Hair removal is an important preparation step to ensure good adhesion of the arrays to the skin surface and should be performed 2 days prior to treatment initiation. Given that follicle density varies according to anatomic location and sex, particular attention should be paid when shaving to ensure adequate adhesion between the arrays and skin, without causing undue damage. A close shave, epilating, or waxing are not recommended due to the risk of skin damage, irritation, and folliculitis. A very short trim with an electric razor is the preferred approach as this will minimize risk of abrasion to the skin and will allow good adhesion of the arrays used to deliver TTFields. Hair removal should be repeated every 7–10 days or as necessary during treatment, depending on speed of hair regrowth, to maximize array adhesion. The razor (or hair-removal implement) should be thoroughly cleaned according to the manufacturer’s guidelines after every use.

##### Managing hyperhidrosis

In the case of hyperhidrosis, aluminum zirconium anti-perspirants can help prevent moisture build-up prior to application of the arrays (41); although it should be noted that it is not known if these agents contribute to electrical impedance. For patients with hyperhidrosis, they should change their arrays more frequently, especially after excessive sweating from exercise.

##### Use of prophylactic topical agents

If prophylactic topical agents are being used, these should be applied with sufficient time to ensure skin penetration, roughly 15–20 minutes, before application of arrays. Any residues should be removed before array placement, by cleaning the skin before being gently patted dry, rather than rubbing to minimize skin abrasion/damage. In general, topical lotions are preferable to creams or ointments as they are less likely to affect electrical impedance.

#### Best practice for array placement and removal

##### Array placement

Hands should be washed carefully before handling the arrays or interacting with the area of application. The arrays are supplied in individual sterile packages to further reduce the risk of infection and should not be used if the packaging is damaged. Due to the innate characteristics of scar tissue, arrays should not be placed over scarred or damaged skin whenever possible.

##### Array removal

Improper removal of arrays could result in skin trauma, increasing the risk of developing skin AEs. Therefore, patients should be provided with clear instructions for changing arrays. Firstly, the adhesive used to hold the arrays in place should be softened using medical grade adhesive or ostomy remover, or warm water (e.g., while showering). Secondly, during showering, the unattached arrays should be peeled off slowly, taking care to avoid any harsh movements. Furthermore, mineral oil (such as baby oil) can be applied to the skin surrounding the array, before slowly peeling back the adhesive patch keeping the array in place, to aid removal (3, 25). Finally, after removal, skin should be thoroughly examined to check for any signs of damage and patients should report any skin damage to a healthcare professional for further monitoring and advice.

##### Array replacement

Consideration should be given to the timing of array application and removal. Healthcare professionals should ensure patients fully understand the application and removal instructions. Patients should be educated on best practices in terms of array changes, which need to be completed every 3–4 days; too often will lead to skin damage, too little will lead to occlusion (3, 42, 43). Before application, the skin should be prepared as previously described; remnant adhesive from prior arrays can be removed by gently wiping the area with mineral oil. Petroleum-based ointments should be avoided due to their impact on electrical impedance (25, 44). New arrays should be applied several hours after removal of the original arrays.

Continuous application of the arrays without proper and regular changes may exacerbate dermatologic AEs. Depending on the location of the tumor, arrays should be repositioned slightly by approximately 2 cm to an alternate layout, allowing any affected skin areas to heal. Alternating layouts, where possible, are important in preventing and treating TTFields therapy-related skin AEs. It should be noted that repositioning the arrays on the torso will still allow for orthogonal fields and maximum targeting of cancer cells.

#### Reducing occlusion and mechanical pressure

There is very little movement in the scalp, however, the thoracic region has greater skin elasticity due to the natural mobility of this region. The skin is required to adapt to twisting, stretching, bending, flexing, and chest wall motion (18). Patients using TTFields therapy in the thoracic region may experience friction between the arrays and the body during normal movement, as well as pressure from the device components when sitting or lying down. This can be mitigated by placing adhesive padding, such as silicone gel or a hydrocolloid dressing, to prevent rubbing and minimize added pressure to the skin, whilst ensuring that adhesion of the arrays is not affected.

During TTFields therapy, physical barriers, such as heavy clothing, and intense exercise should be minimized to reduce the effects of increased temperature and occlusion. Any clothing covering the arrays should allow adequate ventilation to ensure moisture does not exacerbate the effect of occlusion.

### Use of pharmaceutical agents to manage and treat dermatological AEs

Prophylaxis for skin damage is paramount for preventing or reducing the likelihood of developing TTFields therapy-related skin AEs, and a robust prophylactic regimen is imperative as skin AEs may develop in the first 25 days of TTFields therapy initiation. As such, routine prophylaxis with topical steroids or cream calcineurin inhibitors may be warranted in the first 25 days of TTFields therapy, on top of simple skin care techniques to reduce the likelihood of skin AEs from occurring or to reduce the severity of the AE if it occurs, thereby limiting treatment interruption. During treatment planning, consideration should be given to the patient’s individual situation when prescribing either agent as a prophylactic regimen. Consideration of the method and vehicle of application is important; creams, gels, lotions, foams, wipes/pads, sprays, and solutions all demonstrate a low level of TTFields impedance, whilst ointments and petroleum-based products demonstrate high levels of impedance and should be avoided (**Figure 3**) (25). Creams should be applied in a thin layer, allowed to permeate the stratum corneum for 15–20 minutes, and any residue should be removed before applying new arrays. When skin AEs occur, they can be managed effectively in a variety of ways, although prompt intervention is necessary to reduce the need for temporary treatment interruption, which may impact efficacy outcomes.

#### Topical steroids

Topical corticosteroids are commonly available as ointments, creams, gels, or lotions, and less commonly available as sprays, foams, or pastes; healthcare professionals must be mindful about the most appropriate physical properties of topical agents with TTFields therapy. Topical corticosteroids are also available as solutions, which do not reduce the efficacy of TTFields therapy, but may be painful for patients when applied to irritated or shaved skin. Infrequent use of mid-to-high-strength topical steroids in cream, lotion, or solution format such as triamcinolone 0.1% lotion, betamethasone 0.05% cream or foam, fluocinonide 0.05% solution, and clobetasol propionate 0.05% foam or cream are appropriate for symptomatic patients. Additionally, hydrocortisone (not hydrocortisone butyrate) 1% (over the counter, OTC) or 2.5% (prescription only) is a low potency topical steroid that can be used infrequently, for example, once every 2–4 days to coincide with array changes. However, given that topical steroids can cause dermal atrophy with prolonged use, duration of use should be regularly reviewed with the patient according to the Important Safety Information (ISI)/Prescribing Information (PI).

#### Cream calcineurin inhibitors

Topical cream calcineurin and phosphodiesterase 4 (PDE4) inhibitors are nonsteroidal immunomodulators that can be applied to the skin to reduce inflammation. They have a significant advantage over topical steroids as they do not cause dermal atrophy, and are also easily absorbed through the skin, taking 20–30 minutes to permeate through the stratum corneum. Topical calcineurin inhibitors are available as either creams or ointments (45); although the impact of calcineurin inhibitors on electrical impedance has not been specifically tested, it is important that any topical treatment in the form of an ointment is avoided due to the known impact on electrical impedance (25). Additionally, there are limitations with availability, cost, and vehicle selection, which may make them less accessible to some patients.

#### Agents to avoid

Some prophylaxis regimens are not recommended for patients using TTFields therapy. Topical alcohol-based products should be avoided, as they are desiccants and solvents which will irritate the skin, either causing or exacerbating skin AEs such as xerosis and pruritis. Zinc-based creams may reduce the efficacy of TTFields therapy as they are viscous and form barriers (46). In some cases, aluminum chloride formulations used to control sweating can react with sweat to form hydrochloric acid, which is an irritant.

There are many considerations when prescribing prophylactic regimens, including presence or history of existing skin conditions, frequency of application, affordability of chosen therapy, ease of use, ease of prescription, and accessibility for the patient.

### Physical barriers

Skin barriers may be used prophylactically and must be compatible with TTFields therapy. Appropriate skin barriers include: silicone based barriers (such as dimethicone), film-forming polymers in water or organic solvents, and cyanoacrylate formulations (47). Examples of skin barriers that have been tested and demonstrate minimal impact on electrical impedance include SENSI-CARE Sting Free Skin Barrier wipes and SKIN-PREP wipes (25). Petroleum-based skin barriers should be avoided as they have demonstrated a high level of TTFields impedance (25). It should be noted that skin barriers must be removed and reapplied when replacing arrays.

### Management of specific dermatological AEs

#### Dermatitis

Mild-to-moderate strength cream or solution topical steroids are recommended to treat dermatitis as they should not interfere with TTFields therapy efficacy; however, the duration of use should be reviewed and monitored due to the risk of dermal atrophy. Preferred options for TTFields therapy-associated AEs are hydrocortisone (1% OTC or 2.5% on prescription) and triamcinolone acetonide (0.1% or 0.5%). Higher potency corticosteroids, such as clobetasol propionate 0.05%, betamethasone dipropionate 0.05%, or triamcinolone acetonide 0.5% may be considered; however, there is a risk of potential atrophy. The formulation of topical steroids should either be cream, foam, solution, or lotion, to prevent impact on electrical impedance (**Figure 3**) (25).

Alternatively, calcineurin inhibitors or PDE4 inhibitors in cream form can be used to manage dermatitis as they do not cause dermal atrophy and so may be preferential for patients on TTFields therapy with thin or damaged skin. If possible, arrays should not be placed over affected sites and minimized overlap during array exchanges is also recommended.

#### Skin irritation and pruritus

Localized pruritus can be treated with OTC antihistamines, lidocaine, menthol- or pramoxine-based anesthetics, or topical corticosteroids. Refrigeration of these products aids in the antipruritic effect. Arrays should not be placed directly over affected sites.

#### Folliculitis

It is recommended that an antimicrobial cleanser is used in cases of folliculitis (48). When an infection has been confirmed, topical antibiotics such as clindamycin phosphate (foam 1%, gel USP 1%, or lotion) or gentamycin sulfate cream USP 0.1% are advised, as they have minimal impact on electrical impedance (25). Bacitracin zinc may be another appropriate topical antibiotic; however, it has not currently been tested on electrical impedance. If used in solution, topical antibiotics will quickly be absorbed through the skin. Furthermore, they should be used in combination with an antimicrobial cleanser to limit the development of antimicrobial resistance. For severe cases, warm compresses should be used on the affected area, and a course of oral antibiotics may be required. Arrays should not be placed over affected sites.

#### Hyperhidrosis

Hyperhidrosis can be managed using topical aluminum zirconium formulations or topical glycopyrrolate (49). Chloride conjugates, such as aluminum chloride, are not recommended due to the formation of hydrochloric acid in some cases. If excessive sweating is a persistent problem, particularly in warmer climates or during physical activity, anti-sweat, breathable sportswear, or loosely woven materials should be worn, or clothing removed to maximize air circulation. Patients with hyperhidrosis should change their arrays more frequently, especially after activities which may cause excessive sweating.

#### Pressure necrosis

Areas of pressure necrosis should be kept clean to prevent infection, and arrays should not be placed over the affected area. Deep or persistent areas of pressure necrosis may require referral to a dermatologist for evaluation and management.

#### Skin erosions

Once established, erosions can result in further damage to the skin, therefore care should be taken to isolate the affected area. Gently cleaning the wound with minimal rubbing can reduce risk of infection; utilizing silicone tape or hydrocolloid dressing will also minimize rubbing, lowering risk of erosion development and subsequent infection (47). Topical antiseptics which target a range of microorganisms should be considered to cleanse and disinfect wounds. Hibiclens Antiseptic Skin Cleanser is a topical antiseptic agent which has been tested and shows minimal impact on impedance. When infection does present, topical antibiotics should be applied to the affected area. Appropriate options include mupirocin, bacitracin zinc, clindamycin, and gentamycin. Lotion, foam, gel, solution, and cream formulations of topical antibiotics have demonstrated minimal impact on impedance and should be used, whilst ointment-based antibiotics should be avoided (**Figure 3**) (25). Topical and oral antibiotics should be used for more severe cases. Arrays should not be placed directly over affected sites.

#### Skin infections

Antiseptics can be used to clean areas of infection. However, these should only be used when required, and not for routine treatments. Hibiclens Antiseptic Skin Cleanser is a suitable antiseptic option for use in the thoracic region; however, care should be taken regarding the AE profile, as Hibiclens Antiseptic Skin Cleanser should only be applied topically and should not be applied onto the face or scalp. The infected skin area should be swabbed and cultured to identify the causative microorganism, to enable the prescription of an appropriate antibiotic.

#### Topical solution storage and application

For any chosen treatment, application as a spray is preferable to wipes; spray application will create less friction with the skin surface, and therefore should not exacerbate existing AEs. Topical treatments should also be kept cold prior to application, as this will have an antipruritic effect. Arrays should not be placed over affected sites.

## Patient education and support

Educating patients and caregivers on skin care best practices and practical guidance to prevent and reduce TTFields therapy-related AEs is fundamental, given that array changes generally take place outside of the clinic. Education can be provided in a variety of formats.

Clear and simple guidance related to best practice for array changes (placement and removal) should minimize the risk of developing AEs, be easy to follow, and should not cause harm to the treatment area. Fact sheets and infographics could be used to describe and illustrate the appearance of typical TTFields therapy-related dermatological AEs, which would assist patients in knowing when to seek medical advice (**Supplementary Figure 1**). The importance of reporting skin AEs to physicians should also be emphasized. Patients should be encouraged to keep a photo diary of any skin AEs, which could then be referred to during clinic appointments; such information would aid diagnosis and management. Patients could also be provided with a list of OTC dermatologist-recommended agents that are widely available, to ensure patients have a variety of options to prepare and maintain the skin.

Peer-to-peer patient support groups could be encouraged; in addition to enabling patients to meet others using TTFields therapy, it would also allow 1:1 interaction between patients. These groups act as forums to enable open discussions.

## Adapting clinical approaches

Currently there are no standardized tools available for physicians to monitor and record dermatologic AEs in patients receiving TTFields therapy. A standardized tool would enable physicians to assess AEs quickly and accurately and if needed, report findings to the device manufacturer. In addition, skin should be inspected at every appointment to ensure that any irritation is identified as early as possible. An infographic providing a summary on the prevention and management of skin AEs, as well as an interactive multimedia infographic, can be found in the supplementary materials (**Supplementary Figures S1, S2**).

Where possible, specialist nurses could provide support to patients receiving TTFields therapy, either on a 1:1 basis or in a group setting. This support would be accessible to patients ‘on demand’ and would provide reassurance and advice regarding skin AEs. Furthermore, proactive follow ups could help identify skin AEs at an early stage before major intervention is needed. A similar role (wound, ostomy, and continence nurses) already exists for patients with a stoma, which has aided the detection and management of skin AEs, ultimately improving quality of life (50).

## Conclusion

Skin AEs can have a profound effect on a patient’s quality of life during cancer therapy. The skin is a complex organ, and the healing process is multifactorial; skin characteristics vary between individuals and in the different anatomic regions. Therefore, it is important to understand how such characteristics influence the risk of a patient developing TTFields therapy-associated AEs in the thoracic region. Although preliminary analysis has not identified a correlation between select risk factors and incidence of skin AEs, the potential for TTFields therapy-associated AEs to occur as a result of innate and environmental factors should be noted. The PROTECT study (NCT04469075) is currently underway and is investigating the prophylactic effect of topical clindamycin solution 1% and triamcinolone lotion 0.1% in patients with newly diagnosed glioblastoma. Results are likely to provide insights into prevention of dermatologic TTFields therapy-related AEs. Clearer reporting of skin AEs (e.g., timing, severity, and location) will aid analysis and improve understanding of the occurrence of TTFields therapy-associated thoracic skin AEs. A robust prophylactic regimen, adaptation of clinical practice, and patient education may reduce the risk of developing skin AEs; recommendations presented here are a pragmatic approach based on authors’ clinical experiences. In addition, these proactive measures and awareness strategies to improve skin care and prevent/manage skin AEs may prevent treatment interruptions and ultimately support optimization of TTFields therapy usage, which has been correlated with improved survival outcomes.

## Author contributions

MA: Conceptualization, generation of content, writing – review & editing. ML: Conceptualization, generation of content, writing – review & editing. AF: Conceptualization, generation of content, writing – review & editing. ZH: Conceptualization, generation of content, writing – review & editing. JJ: Conceptualization, generation of content, writing – review & editing. BK: Conceptualization, generation of content, writing – review & editing. SK: Conceptualization, generation of content, writing – review & editing. LO: Conceptualization, generation of content, writing – review & editing. RK: Conceptualization, generation of content, writing – review & editing. HA: Conceptualization, generation of content, writing – review & editing. FG: Conceptualization, generation of content, writing – review & editing. All authors contributed to the article and approved the submitted version.

## Supplementary material

Supplementary Figure 1 Infographic on the prevention and management of Tumor Treating Fields (TTFields) therapy-related skin adverse events.

Supplementary Figure 2 Interactive infographic for management and prevention of Tumor Treating Fields (TTFields) therapy-related skin adverse events.
