# Supplementary material for: Expert guidance on prophylaxis and treatment of dermatologic adverse events with Tumor Treating Fields (TTFields) therapy in the thoracic region
Source: Front Oncol. 2023 Jan 4;12:975473. doi: 10.3389/fonc.2022.975473 (PMC9873416; doi:10.3389/fonc.2022.975473)
Supplement: Supplementary Figure 1 — Infographic on the prevention and management of Tumor Treating Fields (TTFields) therapy-related skin adverse events. [file Image_1.pdf]

Prevention and Management of Tumor Treating Fields (TTFields) Therapy-Related Skin Adverse Events

PREVENTION			PROPHYLAXIS	
**Skin health**	**Skin preparation**	**Array placement**	**Topical steroids or calcineurin inhibitor creams**	**Skin barriers**
Routine cleaning and moisturization	Shave with an electric razor 2 days prior to initial array application and repeat as necessary between array changes	Arrays should not be placed over scarred or damaged skin; arrays should be changed at least every 3–4 days; arrays should be repositioned by 2 cm to an alternate layout	Infrequent use of mid-to-high strength topical steroids in cream, lotion, or solution format; calcineurin inhibitors should only be in cream formulation	Skin barriers can be used that are compatible with TTFields therapy, such as SENSI-CARE Sting Free Skin Barrier wipes and SKIN-PREP wipes

Skin adverse event	Advice for mild cases	Advice for severe cases
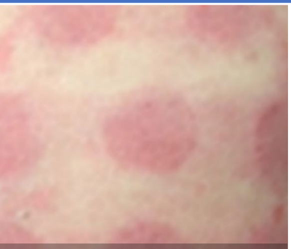 Contact Dermatitis	• Mild-to-moderate strength topical steroid creams or solutions such as:<br>– Hydrocortisone 1% or 2.5%<br>– Triamcinolone acetonide 0.1%<br>• Higher potency steroids may be considered, but may result in dermal atrophy with prolonged use – monitoring is advised if using:<br>– Triamcinolone acetonide 0.5%<br>– Clobetasol propionate 0.05%<br>– Betamethasone dipropionate 0.05%<br>• Calcineurin inhibitor creams or PDE4 inhibitor creams<br>• Consider trimming adhesive if dermatitis occurs due to the tape<br>• Saline compress application in the event of blistering	• Treatment interruption until skin adverse event resolution; restart with prophylactic measures<br>• Possible dermatology consultation
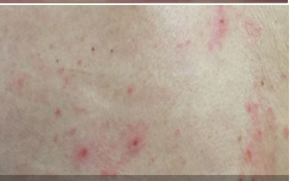 Pruritus	• OTC antihistamines<br>• Lidocaine, menthol- or pramoxine-based anesthetics<br>• Topical steroids (see contact dermatitis)<br>• Refrigeration of topical products will aid in the anti-pruritic effect	• Treatment interruption until skin adverse event resolution; restart with prophylactic measures<br>• Possible dermatology consultation
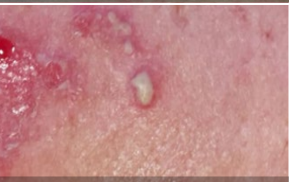 Folliculitis	• Topical antibiotics, in combination with an antimicrobial cleanser, such as:<br>– Clindamycin phosphate solution, gel USP 1%, or foam 1%<br>– Gentamycin sulphate cream USP 0.1%<br>• Ensure electric razors are regularly cleaned to reduce risk of folliculitis	• Application of a warm compress on affected area<br>• Oral antibiotics may be considered<br>• Treatment interruption until skin adverse event resolution; restart with prophylactic measures<br>• Possible dermatology consultation
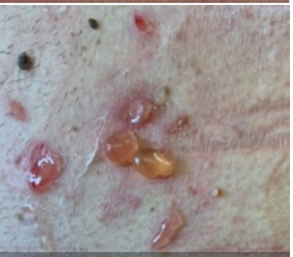 Skin infection	• Antiseptics to clean infected area (e.g., Hibiclens Antiseptic Skin Cleanser)<br>– Antiseptics should not be used routinely or for other types of adverse events<br>– Hibiclens Antiseptic Skin Cleanser can be used on thoracic region, but should not be used on the face/scalp<br>• Topical antibiotics such as:<br>– Clindamycin phosphate solution, gel USP 1%, or foam 1%<br>– Gentamycin sulphate cream USP 0.1%<br>• Avoid direct contact of discs/adhesive tape with affected area(s)	• Take swabs and culture the bacteria to treat with appropriate oral antibiotic<br>• Avoid direct contact of discs/adhesive tape with affected area(s)<br>• Treatment interruption until skin adverse event resolution; restart with prophylactic measures<br>• Possible dermatology consultation
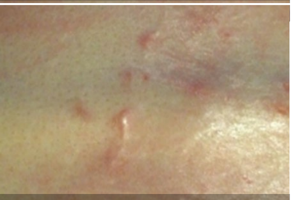 Hyperhidrosis	• Anti-sweat, breathable sports wear, or loosely woven materials are advised<br>• Topical aluminium zirconium formulations or topical glycopyrrolate	
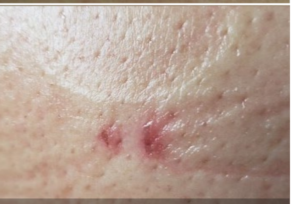 Skin erosion	• Avoid direct contact with affected area(s)<br>• Hibiclens Antiseptic Skin Cleanser may be used to cleanse the wound<br>• Dress affected area with gauze, silicone tape, or hydrocolloid	• If infection is present, consider taking swabs and culturing bacteria and administering appropriate topical/systemic antibiotics<br>• Treatment interruption until skin adverse event resolution; restart with prophylactic measures<br>• Possible dermatology consultation

For severe/persistent cases, interruption of array application for 2–7 days and adherence to guidance is often sufficient

Agents to avoid:

- Any ointments or petroleum-based formulations due to their impact on electrical impedance
- Topical alcohol-based products as they may irritate the skin
- Zinc-based creams as they form barriers which can reduce TTFields therapy efficacy
- Aluminium chloride formulations as there have been cases of hydrochloric acid production

The authors describe generic products consistent with publishing standards. The description of brand/trade names are exclusively limited to instances when specific products were tested.

Novocure® GmbH 2023. All rights reserved.
