# Supplementary material for: Expert guidance on prophylaxis and treatment of dermatologic adverse events with Tumor Treating Fields (TTFields) therapy in the thoracic region
Source: Front Oncol. 2023 Jan 4;12:975473. doi: 10.3389/fonc.2022.975473 (PMC9873416; doi:10.3389/fonc.2022.975473)

Please click on the icons below
to view the relevant content

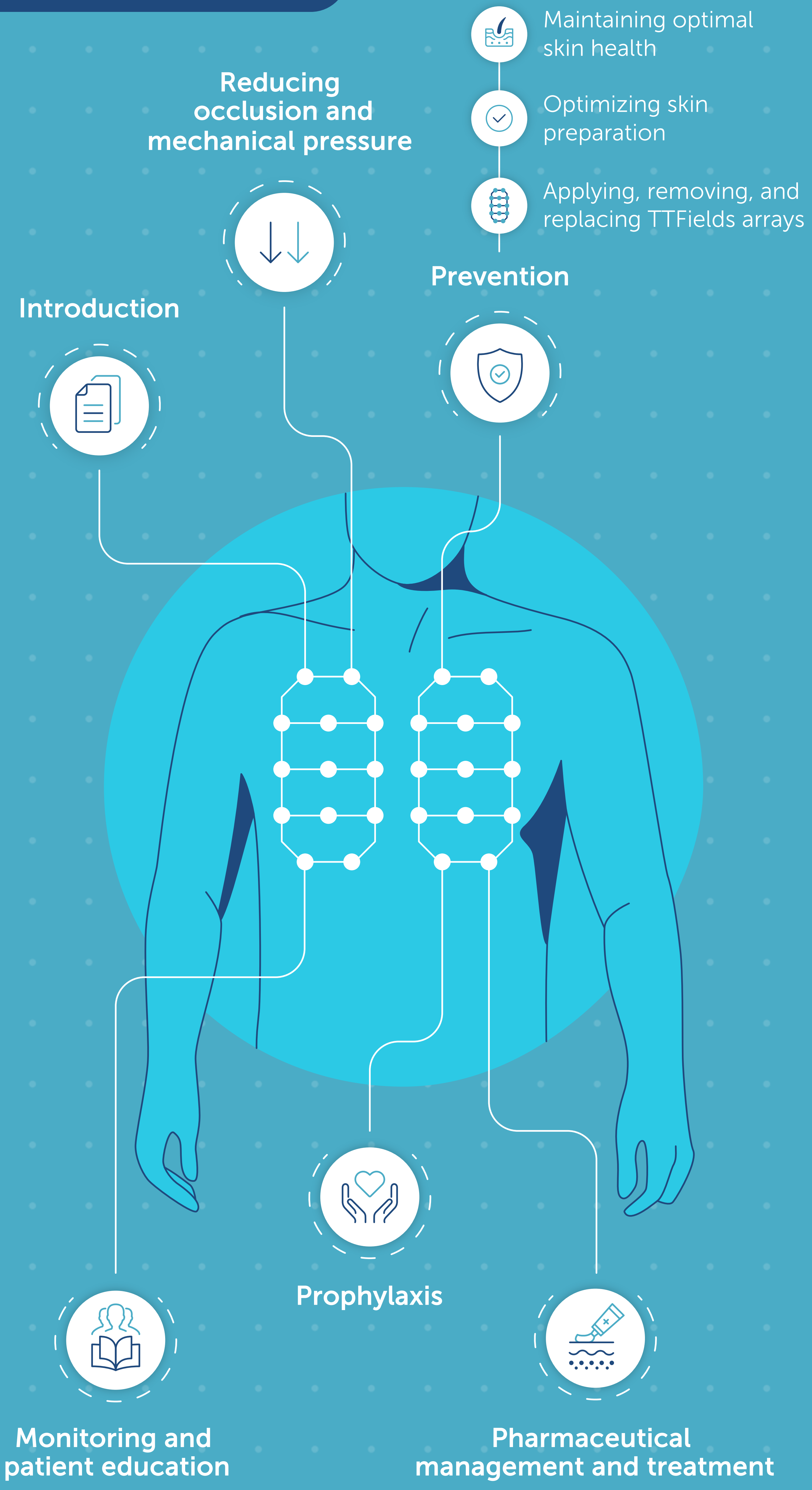

Supplement: Supplementary Figure 2 — Interactive infographic for management and prevention of Tumor Treating Fields (TTFields) therapy-related skin adverse events. [file Image_2.pdf]
